# Influence of dietary fish oil supplementation on DNA damage in peripheral blood lymphocytes of nine healthy dogs

**DOI:** 10.1002/vro2.12

**Published:** 2021-06-22

**Authors:** Francisco J. Pellegrino, Analía Risso, Yanina Corrada, Rocío C. Gambaro, Analía I. Seoane

**Affiliations:** ^1^ School of Veterinary Sciences (FCV) National University of La Plata (UNLP) La Plata Buenos Aires Argentina; ^2^ National Council of Research and Technology (CONICET) Buenos Aires Argentina; ^3^ IGEVET – Institute of Veterinary Genetics “Ing. Fernando N. Dolout” (UNLP‐CONICET LA PLATA), FCV, UNLP La Plata Buenos Aires Argentina

## Abstract

**Background:**

Fish oil (FO) supplementation as a source of omega 3 fatty acids is associated with beneficial effects on health. However, high unsaturated fatty acid content in the diet could result in increased lipid peroxidation and damage to proteins, lipids and DNA. We evaluated the effect of dietary FO supplementation on DNA damage in peripheral blood lymphocytes of dogs. Additionally, we determined the effect of FO supplementation on lipid peroxidation and lipid profile of these dogs.

**Methods:**

Healthy male dogs (*n* = 9) were randomly assigned to one of two diets during 90 days: control (CG, *n* = 4), based on a commercial food, and FO (FOG, *n* = 5), the same food supplemented with 1000 mg FO. Blood samples were collected on days −1, 30, 60 and 90. DNA damage was assessed with the comet assay, and the damage index was obtained. Malondialdehyde (MDA) levels were determined as an indicator of lipid peroxidation. Lipid profile determination included serum triglyceride, cholesterol, low‐density lipoprotein and high‐density lipoprotein levels (HDL).

**Results:**

Damage index values (arbitrary units) were lower in FOG on day 30 (CG, 13.7 ± 2.5; FOG, 6.5 ± 2.5), 60 (CG, 14.7 ± 2.5; FOG, 3.5 ± 2.5) and 90 (CG, 15.5 ± 2.5; FOG, 3.0 ± 2.5) compared with CG (treatment × time interaction, *p* < 0.01). Serum MDA and HDL concentrations were lower in FOG compared with CG on day 60 and 90 (treatment × time interaction, *p* < 0.05).

**Conclusion:**

These findings suggest that dietary FO supplementation did not induce DNA damage in peripheral blood lymphocytes of healthy dogs, but rather reduced it.

## INTRODUCTION

Dietary intake of fish oil (FO) as a source of omega 3 polyunsaturated fatty acids (*n*‐3 PUFA), especially eicosapentaenoic acid (EPA) and docosahexaenoic acid (DHA), is usually associated with beneficial effects on health. In dogs, FO supplementation leads to a rapid increase of EPA and DHA concentrations in plasma^1^ and the incorporation of these fatty acids into blood cell membranes.^2^ However, due to the number of double bonds present in PUFA, a high PUFA content in the diet could result in an increased susceptibility to lipid peroxidation and the generation of a state of oxidative stress.^3^ In lipid peroxidation, free radicals react with the double bonds of unsaturated fatty acids, leading to the formation of thiobarbituric acid reactive substances (TBARS) such as malondialdehyde (MDA).^4^ Previous studies in dogs investigating the effect of FO supplementation on lipid peroxidation markers have yielded conflicting results. For instance, plasma TBARS concentration was found to be increased in old female dogs after 8 weeks of FO supplementation with high *n*‐3 PUFA.^5^ Contrarily, the evaluation of the effect of a diet containing 1.65% FO showed decreased serum MDA concentrations in a group of old male dogs after 8‐week supplementation.^6^ In rats, increased lipid peroxidation in hepatic cells was reported after consumption of an FO‐rich diet for 6 weeks.^4^ Such increase was associated with a suppressive effect of hepatocyte DNA damage, suggesting that FO supplementation would play a protective role against oxidative stress‐induced DNA damage.^4^ However, in a previous study performed by our group, increased DNA damage was observed in peripheral blood lymphocytes of dogs supplemented with FO and subjected to a training programme on a treadmill.^7^

To our knowledge, the effect of dietary FO supplementation on DNA damage in peripheral blood lymphocytes of untrained healthy dogs has not been studied thus far. Therefore, we evaluated such an effect and additionally determined the effect of FO supplementation on the levels of MDA as an indicator of lipid peroxidation, and the lipid profile of these dogs, including serum triglyceride (TG), cholesterol (COL), low‐density lipoprotein (LDL) and high‐density lipoprotein (HDL) levels.

## MATERIALS AND METHODS

### Animals and diets

This study was approved by the Institutional Animal Care and Use Committee (no. 77‐1‐18B) of the School of Veterinary Sciences, National University of La Plata, Buenos Aires, Argentina.

Dogs (*n* = 9) belonged to private owners and were matched by age (5–8 years), sex (all male), height (55–70 cm), body weight (BW) (25–35 kg) and body condition of 3 on a five‐point scoring scale.^8^ Health condition was evaluated with data from complete medical records (history and clinical examination), routine blood and chemical tests and electrocardiograms. Four weeks before the study, dogs were adapted to a standard commercial food according to the regulations established by the Association of American Feed Control Officials fed twice a day (control diet). Meal portions were calculated according to the maintenance energy requirements (MER = 132 kcal × kg metabolic BW [BW^0.75^])^9^ and adjusted to keep the body condition of dogs. Water was given *ad libitum*.

Healthy male dogs were assigned to one of two diets using a computer generated random list. The animals received daily either the control diet (CG, *n* = 4) or the same diet as CG supplemented with a capsule containing 1000 mg FO (FOG, *n* = 5) during an equal period of 90 days. The capsule was administered with the first daily portion. All dog owners gave written informed consent and agreed to feed their dogs with the CG or FOG diet. They also agreed to take their dogs to the School of Veterinary Sciences twice a week to register food intake and body weight of the animals.

The control diet was pooled and analysed for dry matter, neutral detergent fibre, crude protein, lipid and ash according to the Association of Official Analytical Chemists.^10^ Thus, the chemical and energy composition of the control diet on a dry matter basis (DMB) was 28.0% crude protein, 14.0% lipid, 49.1% nitrogen‐free extract, 1.6% neutral detergent fibre, 7.3% ash, 1.4% calcium, 1.0% phosphorus, 0.048% vitamin and mineral mix (2.7 mg vitamin A, 0.045 mg vitamin D3, 60.0 mg vitamin E, 1.0 mg vitamin K, 4.0 mg vitamin B1, 4.0 mg vitamin B2, 4.0 mg vitamin B6, 0.002 mg vitamin B12, 0.6 mg folic acid, 0.5 mg nicotinic acid, 9.2 mg pantothenic acid, 0.1 mg biotin, 400.0 mg choline, inorganic minerals [1.5 ppm calcium iodate, 6.0 ppm iron, 5.0 ppm manganese], chelated minerals [7.5 ppm copper methionine, 0.1 ppm selenium‐enriched yeast, 100.0 ppm zinc propionate]) and 3.7 kcal/g metabolizable energy. Dry matter content was 93.2%. Both diets were analysed by gas chromatography to evaluate their fatty acid composition (Table 1). Samples for lipid extraction were analysed following the method described by Folch, Lees and Sloane‐Stanley.^11^

**TABLE 1:** Fatty acid composition of the control and fish oil‐supplemented diets

Fatty acid	Control diet (%)	Fish oil (%)
Tetradecanoic (C14:0)	0.8	1.0
Palmitic (C16:0)	19.6	14.6
Palmitoleic (C16:1 n‐7)	2.9	6.1
Stearic (C18:0)	4.7	6.1
Oleic (C18:1 n‐9)	25.7	15.6
Linoleic (C18:2 n‐6)	38.1	2.6
Alpha‐linoleic (C18:3 n‐3)	4.0	1.4
Eicosenoic (C20:1 n‐9)	0.1	1.9
Arachidonic (C20:4 n‐6)	1.4	2.2
Eicosapentaenoic (C20:5 n‐3)	0.1	23.2
Docosahexaenoic (C22:6 n‐3)	0.2	13.6
Other fatty acids	2.4	11.7
∑ n‐6	41.3	4.8
∑ n‐3	4.9	38.2

n‐3, omega 3; n‐6, omega 6; n‐7, omega 7; n‐9, omega 9.

### Blood samples

One week before the study (day −1) and on day 30, 60 and 90, venous blood samples were aseptically drawn through venipuncture of the cephalic vein after at least 12‐h fasting. Whole blood was transferred to heparinized tubes (1 ml) and tubes without additive (5 ml). The former were stored at 4°C within 4 h after blood withdrawal until DNA damage analysis. The latter were centrifuged at 1400 x g for 5 min, and serum was recovered and stored at −18°C until lipid peroxidation and lipid profile analysis. The analyses of DNA damage, lipid peroxidation and lipid profile were conducted by blinded independent researchers at the Institute of Veterinary Genetics, School of Veterinary Sciences, National University of La Plata, Buenos Aires, Argentina.

### Single cell gel electrophoresis assay (comet assay)

The comet assay was performed using the alkaline version of the technique previously described^12^ with some modifications.^13^ Briefly, slides were covered with a first layer of 180 µl 0.5% normal agarose (Invitrogen, Carlsbad, CA, USA). Then, 75 µl of 0.5% low melting point agarose (Invitrogen) was mixed with approximately 15000 cells suspended in 25 µl of fresh culture medium and laid onto the slides, which were then immediately covered with coverslips. After agarose solidification at 4°C for 10 min, coverslips were removed, and the slides were immersed overnight at 4°C in fresh lysis solution. The slides were equilibrated in alkaline solution for 20 min. Electrophoresis was carried out for 30 min at 25 V and 300 mA (1.25 V/cm). Afterwards, slides were neutralized by washing three times with Tris buffer (pH 7.5) every 5 min and subsequently washed in distilled water. They were then stained with 1/1000 SYBR Green I solution (Molecular Probes, Eugene, Oregon, USA), analysing 400 randomly selected comet images per treatment.

Scoring was performed at 400X magnification using a fluorescent microscope with a 515−560 nm excitation filter. Based on the extent of strand breakage, cells were classified according to their tail length in five categories, ranging from 0 (no visible tail) to 4 (still a detectable head of the comet but most of the DNA in the tail).^13^, ^14^ Damage Index (DI) was obtained according to Collins^14^ and expressed in arbitrary units, that is, if 100 comets were scored and each comet was assigned a value from 0 to 4, the total score for the sample gel was from 0 to 400 arbitrary units.

### Lipid peroxidation analysis

Lipid peroxidation was assessed with the TBARS assay and reported as serum MDA concentration. The absorbance was detected by spectrophotometry at a wavelength of 532 nm (Multiskan GO, Thermo Fisher Scientific Corporation, Japan).

### Lipid profiles

Serum TG, COL, LDL and HDL concentrations were analysed in a clinical chemistry autoanalyzer (INCCA, DICONEX, Argentina).

### Statistical analysis

Data of the repeated measures over time design were analysed with SAS PROC MIXED (version 9.0; SAS Institute Inc., Cary, NC, USA). Each individual dog was considered an experimental unit. The linear mixed model included the random effect of dog, the fixed effect of time (−1, 30, 60 and 90 days), treatment (CG vs. FOG) and treatment × time interaction. The slice option of SAS was used to detect the time points when significant differences in treatment × time interaction occurred. For the main effect (treatment × time interaction), the alpha level of significance was set at *p* < 0.05. The power of the statistical design of this study was 0.8. It was calculated considering the expected mean in each group, the standard deviation, sample size of each group and a confidence level of 0.95. For example, the expected mean for DNA DI was 15 arbitrary units in CG and 10 arbitrary units in FOG, both with a standard deviation ± 5.0. Sample size was four dogs in each group and a confidence level of 0.95. Values of expected mean and standard deviation were obtained from previous unpublished data from our own laboratory. Descriptive statistics for age, height and BW are reported as mean ± standard deviation. Results of the mixed models are reported as least squares means with standard errors of the mean for an overall treatment group effect and also by time.

## RESULTS

The dog breeds used were Weimaraner (*n* = 2), Boxer (*n* = 3) and mixed breed (*n* = 4). Mean age was 6.2 ± 1.5 years, height at the withers was 60.6 ± 5.3 cm, and BW was 30.2 ± 3.0 kg. Breed distribution was Weimaraner (*n* = 1), Boxer (*n* = 1) and mixed breed (*n* = 2) in CG, and Weimaraner (*n* = 1), Boxer (*n* = 2) and mixed breed (*n* = 2) in FOG.

### Single cell gel electrophoresis assay (comet assay)

**FIGURE 1:**
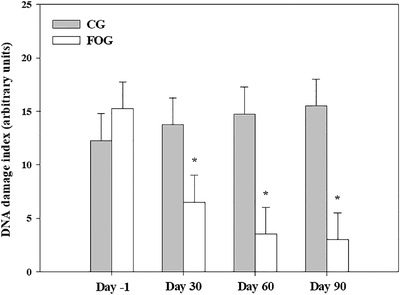
Bar chart showing DNA damage index results (arbitrary units) in nine dogs assigned to the control (CG, *n* = 4) and fish‐oil supplemented (FOG, *n* = 5) groups on day −1, 30, 60 and 90. Values are least squares means and standard error of the means. *Treatment × time interaction, *p* < 0.01

Overall mean DNA DI was lower in FOG compared with CG (overall treatment effect, *p* = 0.03) (Table 2). DNA DI values were lower in FOG compared with CG throughout the study (treatment × time interaction, *p* < 0.01) (Figure 1 and Table 2).

**TABLE 2:** Results of mixed models showing least squares means (LSM, $\bar{X})$ with standard error of the mean (SEM) and *p*‐values for DNA damage index (DI), malondialdehyde (MDA) level as an indicator of lipid peroxidation and the lipid profile levels of serum triglyceride (TG), cholesterol (COL), low‐density lipoprotein (LDL), high‐density lipoprotein (HDL) in the serum overall by treatment group and on day −1, 30, 60 and 90 in nine dogs assigned to the control (CG, *n* = 4) and fish‐oil supplemented (FOG, *n* = 5) groups

	Day −1			Day 30			Day 60			Day 90			Overall treatment effect			
	CG	FOG		CG	FOG		CG	FOG		CG	FOG		CG	FOG		
Variable	$\bar{X}$	$\bar{X}$	SEM	$\bar{X}$	$\bar{X}$	SEM	$\bar{X}$	$\bar{X}$	SEM	$\bar{X}$	$\bar{X}$	SEM	$\bar{X}$	$\bar{X}$	SEM	*p* value
DNA DI (arbitrary units)	12.2	15.2	2.5	13.7	6.5^*^	2.5	14.7	3.5^*^	2.5	15.5	3.0^*^	2.5	14.0	7.0^*^	2.1	0.03
MDA (nM)	137.5	249.2	61.6	211.2	185.0	61.6	274.1	88.7^*^	61.6	244.9	136.9^*^	61.6	213.9	165.0	47.3	0.4
TG (mg/dl)	69.5	89.7	18.0	52.7	71.7	16.6	74.8	53.4	16.0	97.6	72.1	15.5	73.6	71.7	9.4	0.9
COL mg/dl)	217.0	227.5	13.8	223.7	192.0	10.5	226.7	202.0	10.5	227.2	196.0	9.6	223.7	204.3	7.4	0.08
LDL (mg/dl)	39.3	69.2	14.6	76.6	55.3	13.3	82.1	88.9	13.3	69.2	59.5	12.3	66.8	68.2	6.6	0.9
HDL (mg/dl)	130.8	144.2	9.9	137.6	126.0	6.8	143.0	103.7^*^	6.8	142.5	123.7^*^	6.3	138.5	124.4^*^	4.3	0.04

FOG lower compared to CG at these time points *p* < 0.05.

### Lipid peroxidation

While no difference was found in overall mean serum MDA concentration between treatment groups (overall treatment effect, *p* = 0.4) (Table 2), serum MDA concentration was lower in FOG compared with CG on day 60 and 90 (treatment × time interaction, *p* = 0.02) (Table 2).

### Lipid profile

No differences were found in overall means serum TG, COL and LDL concentrations between treatment groups (overall treatment effect, *p* ≥ 0.08) (Table 2). Overall mean serum HDL concentration was lower in FOG compared with CG (overall treatment effect, *p* = 0.04) (Table 2). We did not detect any differences between treatment groups in serum TG, COL and LDL concentrations (treatment × time interaction, *p* > 0.10). Serum HDL concentration was significantly lower in FOG than in CG on day 60 and 90 (treatment × time interaction, *p* = 0.04) (Table 2).

## DISCUSSION

In the present study, we evaluated the effect of dietary FO supplementation on DNA damage in peripheral blood lymphocytes of healthy dogs. Secondarily, we determined the effect of FO supplementation on lipid peroxidation and on changes in the lipid profile of dogs. The results obtained showed that dietary supplementation with 1000 mg FO (232 mg EPA, 136 mg DHA) did not result in DNA damage. By contrast, FOG dogs had lower DI values throughout the 90 days of study compared to the CG dogs. In addition, these lower DI values in FOG were consistent with a lower serum MDA concentration. It is important to note that the values found in all treatments with both comet and TBARS assays were low, meaning they did not imply significant oxidative or cytomolecular damage.^13^

The current data are in agreement with previously reported results showing decreased serum MDA concentrations after 8 weeks of supplementation with 1.65% FO to a group of dogs.^6^ Although diets with high‐PUFA content tend to be associated with higher levels of lipid peroxidation, only one study reported increases in TBARS plasma levels after FO supplementation (EPA, 3.0 g/kg DMB; DHA, 2.6 g/kg DMB) of dogs for 8 weeks.^5^ In another study, serum hydroperoxide concentrations did not differ between groups of young dogs fed with or without FO supplement (EPA, 1.75 g/kg DMB; DHA, 2.2 g/kg DMB).^1^ Differences could be due to the doses used, considering that in the present work the amounts of EPA and DHA administered to dogs were lower than in the mentioned study.^5^ In dogs with osteoarthritis, both FO in a dose of 0.2 ml (EPA, 90 mg; DHA, 20 mg/kg BW) and corn oil supplementation reduced serum MDA concentrations at 16 weeks.^15^ While corn oil does not contain EPA and DHA, the results reported in the mentioned study suggest that it would exert a similar protective effect against lipid peroxidation. Thus, these authors proposed that EPA and DHA from FO could have an antioxidant role or enhance endogenous antioxidant mechanisms.^15^ In our study, DNA DI values were significantly lower in FOG than in CG. Kikugawa et al suggested that the damage caused by hydroperoxides and TBARS, which originate during PUFA peroxidation, would be less potent than the direct DNA damage caused by free radicals.^4^ Thus, this may partly explain the fall of MDA and DNA damage in FO‐supplemented dogs in the current work. Therefore, and in agreement with Kikugawa et al,^4^ dietary FO supplementation would not affect DNA damage. On the contrary, it would be useful to prevent it.

In a previous study, we observed higher DI in peripheral blood lymphocytes of dogs supplemented with FO on day 30 but not on day 60 of a treadmill training program compared with the other studied groups.^7^ These differences could be attributed to the increased production of free radicals in response to physical exercise rather than to higher *n*‐3 PUFA content in the diet. In this sense, the dogs used in that study adapted to exercise intensity, resulting in the absence of DNA damage on day 60.

Regarding changes in lipid profile after FO supplementation, the current results are in line with those reported by Wander et al,^5^ namely, reduced HDL concentration and no effect on TG concentrations. The only difference was concerned with COL values, which were lower in the mentioned report and not affected in our study. On the other hand, Kearns et al reported increased TG and COL concentrations in old male dogs.^6^ Considering that in young dogs TG levels decreased after 12 weeks of FO supplementation.^1^ Differences between the mentioned results could be due to a different metabolic response related to the age of the studied dogs.

One limitation of our study is that dogs belonged to private owners and stayed in their homes during the trial, making the adequate monitoring of dogs difficult. We cannot therefore exclude the possibility of potentially induced exposure or protection from DNA damage.

## CONCLUSION

Dietary supplementation with 1000 mg FO, containing 232 mg EPA and 136 mg DHA, did not induce DNA damage in peripheral blood lymphocytes of healthy dogs, but rather seemed to reduce it. It would be interesting to investigate the existence of a potential *n*‐3 PUFA mechanism to prevent DNA damage.

## CONFLICT OF INTEREST

All authors declare that they have no conflict of interest.
